# Sweat and saliva cortisol response to stress and nutrition factors

**DOI:** 10.1038/s41598-020-75871-3

**Published:** 2020-11-04

**Authors:** Paul Pearlmutter, Gia DeRose, Cheyenne Samson, Nicholas Linehan, Yuqiao Cen, Lina Begdache, Daehan Won, Ahyeon Koh

**Affiliations:** 1 Department of Biomedical Engineering, Binghamton University-State University of New York, Binghamton, NY 13902 USA; 2 Health and Wellness Studies Department, Binghamton University-State University of New York, Binghamton, NY 13902 USA; 3 Department of System Sciences and Industrial Engineering, Binghamton University-State University of New York, Binghamton, NY 13902 USA

**Keywords:** Nutrition, Analytical chemistry, Machine learning

## Abstract

Cortisol is a biomarker for stress monitoring; however, the biomedical and clinical relevance is still controversial due to the complexity of cortisol secretion mechanisms and their circadian cycles as well as environmental factors that affect physiological cortisol level, which include individual mood and dietary intake. To further investigate this multifaceted relationship, a human pilot study examined cortisol concentration in sweat and saliva samples collected from 48 college-aged participants during aerobic exercise sessions along with mental distress and nutrition surveys. Enzyme-linked immunosorbent assays determined highly significant differences between apocrine-dominant sweat (AP), saliva before exercise (SBE), and saliva after exercise (SAE) cortisol concentration (AP-SBE: *p* = 0.0017, AP-SAE: *p* = 0.0102). A significantly greater AP cortisol concentration was detected in males compared to females (*p* = 0.0559), and significant SAE cortisol concentration differences were also recorded between recreational athletes and non-athletes (*p* = 0.044). However, Kessler 10 Psychological Distress Scale (K10) scores, an examination administered to deduce overall wellness, provided no significant differences between males and females or athletes and non-athletes in distress levels, which statistically signifies a direct relationship to cortisol was not present. For further analysis, dietary intake from all participants was considered to investigate whether a multiplexed association was prevalent between nutrition, mood, and cortisol release. Significant positive correlations between AP cortisol, SAE cortisol, K10 scores, and fat intake among female participants and athletes were discovered. The various machine learning algorithms utilized the extensive connections between dietary intake, overall well-being, sex factors, athletic activity, and cortisol concentrations in various biofluids to predict K10 scores. Indeed, the understanding of physiochemical stress response and the associations between studied factors can advance algorithm developments for cortisol biosensing systems to mitigate stress-based illnesses and improve an individual’s quality of life.

## Introduction

Cortisol is a glucocorticoid hormone produced in the adrenal cortex of the hypothalamic–pituitary–adrenal (HPA) axis that is responsible for assessing adrenocortical function^1^, immune response mechanisms^2^, both cardiovascular and central nervous system processes^3^ and regulating glucose metabolism by antagonizing insulin production^4^. The most significant effect on basal cortisol concentration variation stems from psychological and physiological stressors^5^. Abnormal cortisol secretion levels, which depend on the time of day due to its adherence to the body’s circadian rhythm, can compromise the immune system by preventing inflammation pathways, which leads to debilitating conditions such as Addison’s disease (hypocortisolism) and Cushing’s disease (hypercortisolism)^6^. Nutrition and cortisol secretion levels are closely linked, as previous research studies have found correlations between stressors and obesity^7^. Unhealthy dietary habits are connected to HPA axis hyperactivity, leading to oversecretion of basal cortisol and large-scale fat accumulation^8^. Moreover, the consumption of unhealthy foods, such as high carbohydrate or high-fat foods, is seen as rewarding during periods of undergoing stress, but can elevate cortisol levels by progressively increasing stress levels^8^.

Recognizing differences between the various bodily cortisol concentration levels is critical, as they are the primary glucocorticoid biomarkers in an organism's homeostatic response to stress^9^. Serum and saliva cortisol detection methods have been researched mainly in previous studies for biomedical and psychological applications^2,10,11^. Saliva has proven more lucrative for cortisol determination due to its non-invasive in situ collection method, and its high correlation values to cortisol concentration levels in serum^11^. Unbound free cortisol in saliva is found at much lower concentration levels than serum, as 14% of salivary cortisol is bound to cortisol binding globulin (CBG) and 30% of free salivary cortisol is enzymatically transformed into cortisone^11^. Salivary cortisol levels have previously been reported to range between 10.2–27.3 ng/mL in the morning and 2.2–4.1 ng/mL at night for healthy adults^12^. Sweat is another less common biofluid source that can be used for non-invasive cortisol detection. Cortisol can be obtained from apocrine or eccrine sweat, which is secreted from glands found on hair follicles or secreted from glands located on the surface area of the skin, respectively^1^. Sweat cortisol analysis can be advantageous to use over saliva because it can be collected continuously by wearable devices with minimal human intermediation^13^. Recently, emerging wearable biosensors have allowed in situ sweat cortisol detection, observed correlations between sweat and saliva cortisol concentrations, and observed positive correlation post-induced sweat intervention^14^. However, these wearable sensing systems need further optimization by validating an acceptable sweat cortisol range, as previous studies have reported conflicting cortisol concentration results^13,15^.

Herein, we conducted a human pilot study to assess the associations between dietary choices, effects of stress on overall mood, cortisol secretion in collected biofluids, and the capability for prediction of stress levels. Sweat and saliva cortisol concentration ranges were determined for the study participants, which further contribute to the accepted values and differences of cortisol levels in these biofluids. Sex factors and athletic activity were incorporated as variables in the study for comparable nutrition, mood, stress, and cortisol concentration assessments. This work highlights the complicated relationships between the varying factors that contribute to elevated cortisol secretion levels in sweat and saliva and development of a stress prediction model via machine learning.

## Results

### Quantification of mood, stress and diet

Table 1 summarizes the results of the Kessler Psychological Distress Scale (K10) surveys, Mood and Anxiety Symptoms Questionnaires (MASQ), nutritional intake, and general information taken from the participants in the study. Further breakdown of the age-ranges of all participant subgroups is displayed in Supplementary Table S1. All statistical testing made between males (n = 18), females (n = 30), recreational athletes (n = 22) and non-athletes (n = 26) was with the two-tailed Mann–Whitney U test. Significant differences in height, weight, body mass index (BMI), basal metabolic rate (BMR), and protein consumption were reported between male and female participants (*p* < 0.05). Males were significantly taller (M: 174.7 ± 6.4 cm, F: 164.8 ± 7.0) and heavier (M: 80.6 ± 10.3 kg, F: 64.4 ± 13.2 kg), required more caloric intake (M: 1886.0 ± 138.8 cal/day, F: 1459.2 ± 138.3 cal/day), and consumed more protein than females (M: 334.9 ± 133.6 g, F: 198.4 ± 71.3 g) in this study. However, no significant differences were present in calories consumed per day/ BMR (Cal Per Day/BMR) between males (1.2 ± 0.5) and females (1.1 ± 0.3), as both sexes met their basic caloric needs on average. Alternative Healthy Eating Index (AHEI) scores (M: 21.8 ± 10.2, F: 19.6 ± 10.2) indicated that a majority of males fulfilled between one to three recommended daily amounts for the five food groups and a majority of females fulfilled one to two of the recommended daily amounts for the five food groups in their diets.

**Table 1:** Demographic information, mood and stress scores, and nutritional intake for study participants.

	Male (n = 18)	Female (n = 30)	p^s^	Athlete (n = 22)	Non-Athlete (n = 26)	p^a^
Age (y)	23.1 ± 4.0	21.8 ± 2.6	n.s	21.1 ± 2.9	23.3 ± 3.1	< 0.001
Height (cm)	174.7 ± 6.4	164.8 ± 7.0	< 0.00001	169.4 ± 9.4	167.7 ± 7.3	n.s
Weight (kg)	80.6 ± 10.3	64.4 ± 13.2	< 0.0001	71.3 ± 13.8	69.8 ± 15.1	n.s
BMI (kg/m^2^)	26.4 ± 2.9	23.6 ± 4.1	< 0.05	24.8 ± 4.0	24.6 ± 3.9	
K10 Score	18.7 ± 6.0	20.2 ± 5.9	n.s	19.1 ± 4.8	20.1 ± 6.8	n.s
MASQ Day 2 Score	64.2 ± 10.8	61.9 ± 12.3	n.s	61.2 ± 12.9	64.0 ± 10.7	n.s
*MASQ Day 5 Score (n = 46)	61.4 ± 10.3	58.3 ± 9.6	n.s	56.2 ± 8 .9	61.9 ± 10.0	< 0.05
*Birth control users (n = 12)	0	12	N/A	7	5	N/A
BMR (Cal/day)	1886.0 ± 138.8	1459.2 ± 138.3	< 0.00001	1608.6 ± 232.7	1628.3 ± 267.6	n.s
*Calories per day/BMR (n = 45)	1.2 ± 0.5	1.1 ± 0.3	n.s	1.2 ± 0.3	1.1 ± 0.5	n.s
*AHEI (n = 44)	21.8 ± 10.2	19.6 ± 10.2	n.s	18.1 ± 8.7	22.6 ± 11.0	n.s
*Simple carbs (g) (n = 44)	513.2 ± 265.9	434.9 ± 152.9	n.s	523.4 ± 207.0	412.0 ± 191.9	< 0.1
*Complex carbs (g) (n = 44)	157.0 ± 126.8	148.6 ± 116.4	n.s	163.1 ± 136.0	141.6 ± 103.5	n.s
*Sugar (g) (n = 45)	204.2 ± 116.9	180.5 ± 74.1	n.s	212.1 ± 77.4	169.6 ± 100.6	n.s
*Protein (g) (n = 45)	334.9 ± 133.6	198.4 ± 75.3	< 0.001	249.4 ± 94.4	250.5 ± 141.0	n.s
*Fat (g) (n = 45)	241.7 ± 106.2	191.2 ± 71.3	n.s	238.6 ± 78.5	181.5 ± 90.9	< 0.05

The values signify mean ± standard deviation. Carbs = Carbohydrates, n.s. = non-significant, *p*^s^: differences between males and females (Mann–Whitney U test), *p*^a^: differences between athletes and non-athletes (Mann Whitney U test). Categories with a * signify where not all 48 participants provided recorded information.

Significant differences in age, Day 5 MASQ scores, simple carbohydrate consumption, and fat consumption were found (*p* < 0.1) between athletes and non-athletes. Athletes were significantly younger (A: 21.1 ± 2.9, NA: 23.3 ± 3.1), exhibited lower negative mood scores for the morning of the exercise session (A: 56.2 ± 8.9, NA: 61.9 ± 10.0), consumed more simple carbohydrates (A: 523.4 ± 207.0 g, NA: 412.0 ± 191.9 g), and consumed more fat than non-athletes (A: 238.6 ± 78.5 g, NA: 181.5 ± 90.9 g). Athletes, based on AHEI scores, consumed only recommended daily amounts of one to two of the five food groups, whereas non-athletes exhibited healthier eating patterns and consumed one to three of the recommended daily amounts of the five food groups (A: 18.1 ± 8.7, NA: 22.6 ± 11.0). Both groups met their basic daily caloric needs, on average, as Cal Per Day/BMR ratios were above 1 (A: 1.2 ± 0.3, NA: 1.1 ± 0.5).

For each group, the average K10 score ranged from 18.7 to 20.2, which portrays likely wellness on the verge of mild stress for the average study participant. MASQ score values decreased from Day 2 to Day 5 significantly (two-tailed Wilcoxon signed-rank test, *p* = 0.01078), which indicates an increase in positive mood for participants who provided responses to both MASQs (n = 46) as the week progressed.

### Biofluid cortisol concentration analysis

For this study, not all biofluid data for every participant was able to be adequately quantified. A hurdle that prevented the collection of sweat samples was that little to no volume of sweat was extracted during the centrifugation process for certain participants. To avoid skewing cortisol levels, participants were not pressured to exceed exercise intensity to obtain enough sweat for analysis because the physical activity can induce stress response. Cortisol concentrations in biofluids were determined and accepted for statistical analysis when higher than each ELISA kit’s limit of detection (LoD) of 0.47 ng/mL, and 0.5 ng/mL, for sweat and saliva samples respectively. Table 2 provides information on the total number of samples and comparable samples that were studied for each biofluid.

**Table 2:** Collected biofluid samples overview.

	Apocrine-dominant armpit sweat (AP)	Eccrine-dominant lower back sweat (LB)	Saliva before exercise (SBE)	Saliva after exercise (SAE)
Total samples	25	7	47	40
Males	8	3	17	16
Females	17	4	30	24
Athletes	15	5	21	20
non-athletes	10	2	26	20
average cortisol (ng/ml)	2.40	1.21	5.70	5.27
Standard deviation (ng/mL)	2.33	0.49	6.35	6.71
Range (ng/mL)	0.64–10.84	0.70–1.88	0.65–26.40	0.50–30.72
Comparable AP, LB, SBE, and SAE samples				4
Comparable AP, SBE, and SAE samples				23
Comparable SBE and SAE samples				39

**Figure 1:**
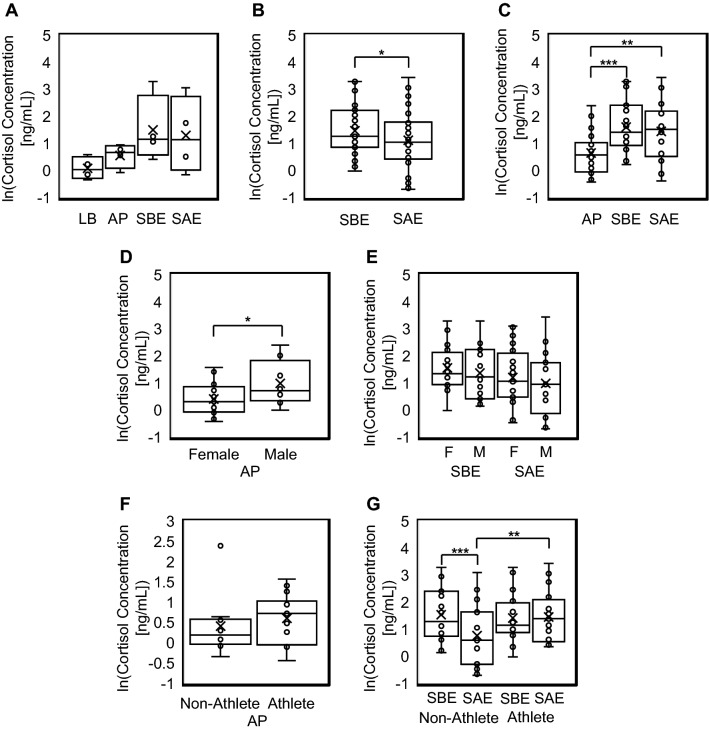
Comparison of natural log transformed cortisol concentrations in collected biofluid samples. (**A**) Comparable LB, AP, SBE, SAE samples (n = 4) (**B**) Significant cortisol decrease (two-tailed paired t test, *p* = 0.0597) between total comparable SBE and SAE samples. (n = 39) (**C**) Significant difference between total comparable (n = 23) AP, SBE, and SAE samples (one-way ANOVA, *p* = 0.0012). Tukey-HSD post hoc analysis details significant difference between AP-SBE (p = 0.0017) and AP-SAE (p = 0.0102). (**D**) Significant increase in AP male (n = 8) cortisol levels compared to AP female (n = 17) cortisol levels (two-tailed independent t test, *p* = 0.0559). (**E**) No significant differences present between female (n = 24) and male (n = 15) SBE-SAE samples (one-way ANOVA, *p* > 0.1). (**F**) No significant difference between non-athlete (n = 10) and athlete (n = 15) AP cortisol concentrations (two-tailed independent t test, *p* > 0.1). (**G**) Comparable SBE-SAE samples of non-athletes (n = 19) show significant decrease of cortisol (two-tailed paired t test, *p* = 0.005). Significant increase of SAE cortisol levels in athletes (n = 20) compared to non-athletes (two-tailed independent t test, *p* = 0.043).

Only four comparable sweat, eccrine-dominant lower back (LB) and apocrine-dominant armpit (AP), and saliva samples, saliva before exercise (SBE) and saliva after exercise (SAE), were able to be quantified. Meaningful statistical significance could not be analyzed between all compared biofluids (Fig. 1A) due to the limited amount of collected comparable data samples. AP, SBE, and SAE samples were all able to be further quantified for comparison testing. As shown in Fig. 1, statistically significant differences were found between different groups and different biofluids. Supplementary Fig. S4 displays the different biofluid cortisol ranges between subgroup populations, but conclusions derived from these data are inherently limited due to the smaller sizes of these groups. For the comparable SBE (6.48 ± 6.69 ng/mL) and SAE (5.42 ± 6.73 ng/mL) samples collected (n = 39), cortisol concentration in saliva significantly decreased after exercise (two-tailed paired t test, *p* = 0.0597). However, the inherent workout intensity affected changes in cortisol concentration in saliva between non-athlete and athlete groups (Fig. 1F), as non-athlete (SBE: 6.87 ± 6.75 ng/mL, SAE: 4.16 ± 5.62 ng/mL) comparable SBE-SAE samples (n = 19) revealed a significant decrease in cortisol levels (two-tailed paired t test, *p* = 0.005), while athlete (SBE: 6.12 ± 6.78 ng/mL, SAE: 6.62 ± 7.60 ng/mL) comparable SBE-SAE (n = 20) cortisol levels displayed no significant difference. SAE samples from athletes showed a significant increase from non-athletes (two-tailed independent t test, *p* = 0.043). However, when analyzing raw AP cortisol concentration levels, non-athletes exhibited an average range (2.79 ± 3.45 ng/mL, n = 10) that is non-significantly different than athletes’ range (2.13 ± 1.22 ng/mL, n = 15). After the natural logarithmic transformation of the AP dataset, due to presence of outliers and skewed data, athletes still portrayed cortisol levels (ln[AP]: 0.60 ± 0.60) that were non-significantly different to non-athletes (ln[AP]: 0.40 ± 0.77).

A one-way analysis of variance (ANOVA) test between total comparable AP (2.52 ± 2.40 ng/mL), SBE (7.46 ± 7.67 ng/mL), and SAE (6.93 ± 7.74 ng/mL) samples (n = 23) shows significant difference between the groups (*p* = 0.0012) (Fig. 1B). Tukey-HSD post hoc analysis revealed there was a significant difference between apocrine-dominant armpit sweat and saliva (AP-SBE: *p* = 0.0017, AP-SAE: *p* = 0.0102). When analyzing a possible difference in cortisol levels between sexes, female AP cortisol levels (1.77 ± 1.19 ng/mL, n = 17) were significantly lower than male AP cortisol levels (3.73 ± 3.53 ng/mL, n = 8) (two-tailed independent t test, *p* = 0.0559). However, no significant differences were found between male (SBE: 6.09 ± 6.75 ng/mL, SAE: 5.29 ± 7.77 ng/mL, n = 15) and female (SBE: 6.73 ± 6.78 ng/mL, SAE: 5.50 ± 6.18 ng/mL, n = 24) SBE-SAE cortisol concentrations (Fig. 1D).

### Comparison of cortisol to stress levels and diet

**Figure 2:**
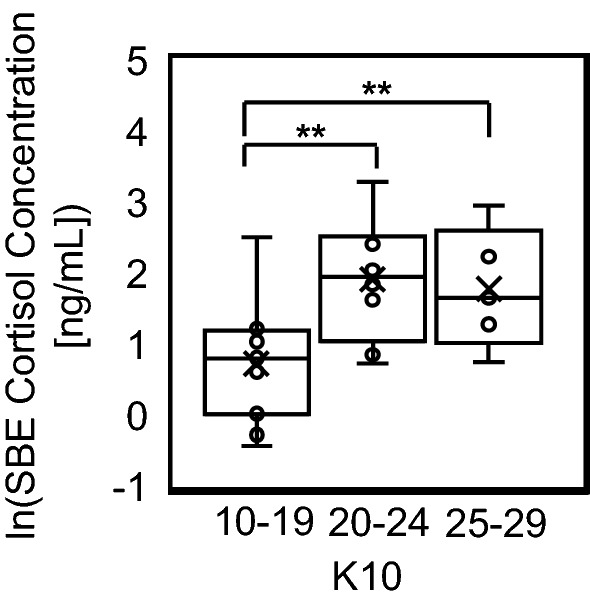
Comparison of non-athlete ln(SBE) between low (10–19), mild (20–24), and moderate (25–29) mental distress K10 groups. Kruskal–Wallis testing showed significant difference (*p* = 0.02244) between the groups. Post hoc Mann–Whitney U testing showed significant increase in cortisol concentration range was reported between low-mild (*p* = 0.01878) and low-moderate groups (*p* = 0.04136).

Biofluid cortisol concentrations were analyzed with recorded K10 stress scores to assess if possible correlations between both variables exist. For total AP, SBE, and SAE biofluid populations, no significant differences were found between any of the K10 mood score groupings (Supplementary Fig. S4). These non-significant findings were in accordance with the analysis of K10 as the lone variable between sexes and athletic activity groups, as no significant differences were found between them as well (Table 1). When further analyzing the connection between K10 scores and biofluid cortisol, a significant difference was found between the low (n = 11), mild (n = 8) and moderate (n = 5) mental distress groups for non-athlete SBE samples (Fig. 2). Because this was the only found dataset that visualized this correlation, additional analysis had to be made with mood scores and nutritional factors to deduce the complexity of both their relationship with cortisol concentration levels.

**Figure 3:**
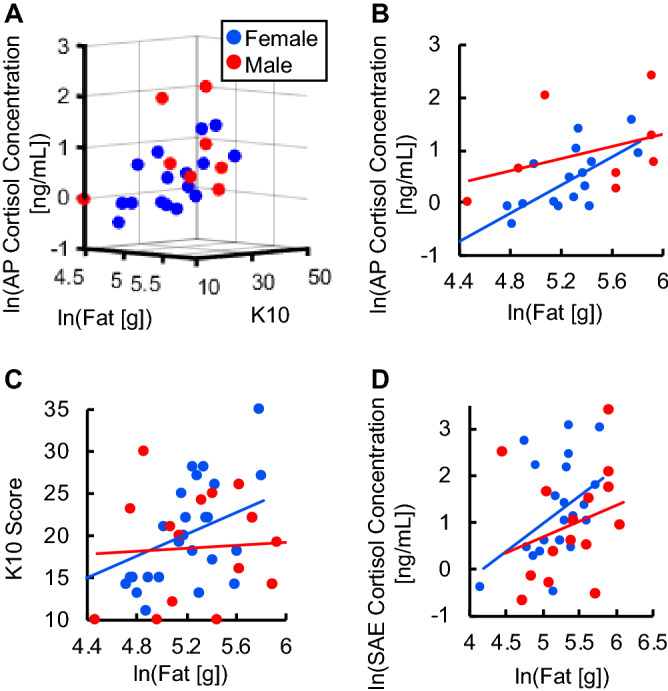
Correlations in fat consumption, K10 scores and biofluid cortisol levels for males and females. (**A**) Three-dimensional scatter plot for ln[fat], K10, and ln[AP]. Comparison of males and females between ln[fat] and (**B**) ln[AP] (female R^2^ = 0.4451, linear regression ANOVA: *p* = 0.0048) (**C**) K10 (female R^2^ = 0.1994, linear regression ANOVA: *p* = 0.017) (**D**) ln[SAE] (female R^2^ = 0.1797 , linear regression ANOVA: *p* = 0.049).

Biofluid cortisol concentrations in relationship to K10 scores and nutritional factors were further investigated to assess any correlations within the study groups. Figure 3A displays a three-dimensional scatter plot for AP cortisol, K10 scores, and natural logarithm of fat consumption for male (n = 8) and female (n = 16) participants. This figure seemingly visualizes a positive correlation between fat consumption, K10 scores and AP cortisol levels in female participants. This plot was further broken down into a two-dimensional fat intake versus AP cortisol graph (Fig. 3B) and a two-dimensional fat intake versus K10 (n = 30) score graph. (Fig. 3C) There is a positive correlation between fat and AP cortisol levels at R^2^ = 0.4451, with a significant linear regression ANOVA, *p* = 0.0048. A positive correlation between fat intake and K10 scores was also present at R^2^ = 0.1994 and significant linear regression *p* = 0.017. Significant positive correlation for linear regression was also found between fat intake and SAE cortisol levels (n = 24) at R^2^ = 0.1797 and an ANOVA, *p* = 0.049 (Fig. 3D).

**Figure 4:**
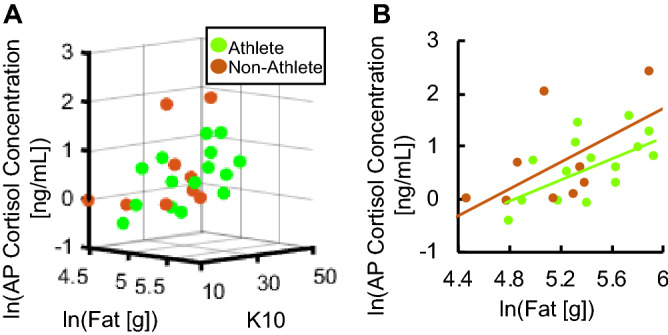
Correlations in fat consumption, K10 scores and biofluid cortisol levels between athletes and non-athletes. (**A**) Three-dimensional scatter plot for ln[fat], K10, and ln[AP] for athletes and non-athletes. (**B**) Comparison between ln[fat] and ln[AP] for athletes and non-athletes at R^2^ = 0.3568 for athletes. (Linear Regression ANOVA: *p* = 0.019).

A three-dimensional scatter plot was also constructed for athletes (n = 15) and non-athletes (n = 10) that compares AP cortisol levels, K10, and fat consumption. (Fig. 4A) This figure showed a possible correlation between fat intake amount and AP cortisol concentrations for athletes that was further corroborated in Fig. 4B. with a R^2^ = 0.3568 and a significant linear regression ANOVA, *p* = 0.019. Other nutritional intake factors, which include simple and complex carbohydrates, sugar and protein consumption did not show correlation with cortisol concentration and K10 scores.

Based on the constructed three datasets from the machine learning framework (see method section), evaluation of the performances by the mean absolute error (MAE) and its standard deviation through the leave-one-out cross-validation method was made. Supplementary Fig. S5 illustrates all prediction results with respect to the actual K10 score. Due to the heterogeneity of the input data, a variation of the prediction error exists across the sample population. For instance, the model KNN could exactly predict the K10 score for the sample ID C-025, whereas there is a large gap between the prediction and actual stress level on the sample ID C-014. Thus, measurements for not only the absolute error of each sample individually but also calculate the standard deviation across all errors to represent the robustness of the prediction models. All results are summarized in the following Table 3. The KNN can predict the K10 score with respect to the given information with an error rate of 3.60, which outperforms the other algorithms. Moreover, the standard deviation from the KNN shows the best prediction performance compared to other methods except for the case of SBE samples (n = 48) dataset. In terms of the prediction error across the dataset, AP cortisol level is observed as the most beneficial way to predict the K10 score without significant change over the variation.

**Table 3:** Summary of statistical difference between actual and machine learning results.

	Mean absolute error			STD		
	Saliva		Armpit	Saliva		Armpit
Number of data	48	28	28	48	28	28
Random forest	5.83	5.82	4.39	3.07	3.50	3.71
Decision tree	5.22	4.54	4.82	3.53	3.42	3.55
Ordinal regression	3.50	3.92	4.34	2.26	2.73	3.01
KNN	4.10	3.62	3.60	3.57	2.27	2.24

## Discussion

This study’s nutritional analysis suggests that unhealthy eating behaviors among college-aged individuals are potentially significant contributors to stress accumulation. Previous research has demonstrated that college students face barriers, such as convenience of unhealthy food and stress factors, that prevent healthy eating habits^17^. Using the AHEI as a benchmark for proper nutrition, not one participant in the study fulfilled the all daily recommended values for the five food groups. The disparity between what dietary habits are considered health-conscious and the nutritional habits of the study group, nutrition was deliberately analyzed as a factor for stress examination. Poor nutritional intake, daily stressors and stress induced by endurance exercise may have contributed to the variation of cortisol secretion levels for participants in this study.

Salivary cortisol provided insightful, but some unexpected, results due to the total decrease in concentration levels post-exercise. The significant saliva cortisol decreases among non-athletes, and lack of any significant saliva cortisol difference among athletes for the exercise study session, contradicted the initial hypothesis that both groups’ cortisol levels would increase post-workout. This difference in results can be attributed to two possible factors: the cortisol awakening response (CAR) and the intensity of individual participant’s exercise. The CAR refers to free cortisol levels that rapidly increase after awakening as an endocrine response to subtle changes in hypothalamus–pituitary–adrenal (HPA) axis activity^18,19^. This initial spike in cortisol levels has a delayed drop off over time throughout the course of the morning, which would have occurred during the workout session^18,19^. In regard to exercise activity, low to moderate intensity workout sessions, at time periods under 40 min, have shown no significant differences in salivary cortisol concentration in past studies^20^. In a previous study conducted by Hill et al., significant spikes in cortisol levels were present only when participants have reached 60–80% of their maximal oxygen uptake (VO_2max_)^18^. With these known factors, athletes were determined to have exercised at a moderate intensity while non-athletes exercised at a low intensity for the duration of the session. This difference in workout intensity sustained athletes’ cortisol levels compared to their non-athletic counterparts. Past research revealed inconclusive evidence of any significant differences between athlete and non-athlete salivary cortisol levels during exercise^21^, which signifies more controlled data must be collected to contribute further analysis for these groups. The lack of assessed significant difference in salivary cortisol between sexes is similar to reports by Wüst et al., where only 3% of saliva cortisol variability was reported due to sex differences^16^.

Due to sweat’s limited past usage as an experimental cortisol detection tool, it was difficult to base this study’s results on any previous findings. Only one participant provided sweat cortisol levels within the previously accepted normal range of 8–141 ng/mL^15^. This range was in accordance with published data by Jia et al., who had previously reported an average sweat cortisol concentration range of 0.5–1.68 ng/mL for their participants, which matches more similarly to the range of cortisol concentration values reported from this study^13^ The significantly higher expression of apocrine-dominant sweat cortisol in males compared to females has not been extensively documented. However, these results do agree with a study done by Tu et al., where male apocrine sweat displayed significantly higher corticosteroid reaction levels than female apocrine sweat^22^. This phenomenon can be explained by the pituitary gland’s divergence in development between males and females post puberty, as it is the primary regulator of cortisol secretion^23^. The concentration range of eccrine-dominant cortisol determined was comparable to that reported by Jia et al., but the analyzed apocrine-dominant sweat range fell between Jia et al.’s eccrine sweat cortisol range and the accepted physiological range for sweat cortisol. The reported apocrine-dominant sweat cortisol concentration values can be applicable for future studies, as previous ranges were reported only for eccrine sweat. Therefore, comprehensive sweat information from both apocrine and eccrine glands enables the creation of accurate sweat-based biomedical diagnosis systems. With more sweat apocrine and eccrine sweat collected, increasing evidence can be provided for establishing the accepted sweat cortisol concentration ranges. Future studies can engage in collecting more comparable apocrine and eccrine sweat samples using various sweat inducing approaches (e.g., iontophoresis and heat-induced sweat) to make more concrete statistical analysis between the different non-invasive biofluid cortisol concentration ranges. With higher amounts of sweat cortisol data made available, sweat can become an industry standard cortisol determination method rather than just an alternative method for stress hormone dynamic quantification^24^.

Direct comparisons between either MAQS mood scores or K10 distress scores to biofluid cortisol concentrations provided minimal correlation. MAQS surveys provided no significant direct correlation to any biofluid cortisol concentrations or nutritional consumption habits. K10 scores provided minimal evidence for direct relationships with cortisol secretion levels with significant differences being present only between low and mild/moderate stress groupings for SBE samples of non-athletes. The results showed the potential for directly correlating K10 scores with general saliva cortisol levels, as those who present themselves with mental distress have statistically significantly higher cortisol levels than those who appear to be mentally well. However, no supporting statistically acceptable evidence was found for athletes’, males’ and females’ SBE, SAE, and AP cortisol concentration levels correlating with K10 stress groupings in this study. With little other supporting evidence found in this study, further analysis in future experiments can provide stronger support for the claim that these K10 distress scores are directly linked to basal cortisol release. The multi-dimensional positive correlations between fat consumption, worsening overall stress, and increasing cortisol levels in females and athletes proved to be significant, which confirms significant positive correlations between fat, K10 scores, and AP/SAE cortisol concentration levels of females and athletes. Athletes’ consumed significantly higher amounts of fat in their diets when compared to non-athletes, which was potentially a factor linked to their increased cortisol response during exercise. The biological relationship between these variables is attributed to excess fat accumulation that propels the hyperactivity of the HPA axis^8^. Fatty food consumption can be perceived as gratifying during periods of stress but also an aid to increased HPA axis activity^8^. Cortisol’s role as a regulator for fat metabolism supports these positive correlations found in females and athletes as well^2^. Collecting more samples and including various factors (e.g., tobacco, steroid/hormonal drugs) in future experiments for additional analysis can be beneficial, as other potential multiplexed correlations can be made for stress determination.

To further advance the application of the study, the predictive methods based on various machine learning models, which are highly capable of handling the nonlinear and heterogeneous relationship between the collected variables and the stress level, K10 score were studied. The outcome of the prediction modeling indicates that the K10 score can be predictable with ~ 3.5 error rate. A variation of the performance across the different samples (approx., 3.2) suggests to an in-depth study for individual-level analysis with big-data set in the future.

## Conclusion

This human pilot study contributes to knowledge of biofluid cortisol concentration, its differences between sexes, and nutrition’s effect as a variable in stress determination. The significant differences established between male and female sweat cortisol helps establish the different normalized ranges for sweat cortisol concentration levels. Saliva cortisol activity between athletes and non-athletes in response to exercise stimulation shows significant difference. The three-dimensional positive correlations between fat intake, K10 scores, and AP/SAE cortisol in females and athletes proved significant, as cortisol was unable to be a direct indicator of stress levels. The known biological associations between fat accumulation and HPA axis overactivity support the positive correlations found between stress, cortisol, and fat intake in the study. With the large assortment of variables analyzed, stress determination cannot be made with a single factor, such as cortisol secretion levels alone, but with collective data analysis.

## Methods

### Materials and instrumentation

Sweat was collected using sterilized medium gauze pads (Johnson & Johnson, 7.62 cm $\times$ 30.48 cm) and absolute waterproof tape (Nexcare). Standard urinary cortisol ELISA (0.47–200 ng/mL range) and saliva cortisol (0.5–100 ng/mL) ELISA kits, for sweat and saliva cortisol analysis respectively, were purchased from Eagle Biosciences (Amherst, NH). Methanol (99% ACS grade) was purchased from VWR (Atlanta, GA). Artificial sweat was purchased from Pickering Test Solutions (Mountain View, CA). Cortisol solution in methanol at 1 mg/mL was purchased from MillporeSigma (Burlington, MA). Deionized water is purified (18.2 MΩ cm; total organic content < 6 ppb) using a Millipore Milli-Q Gradient A-10 purification system (Bedford, MA). Spectroscopic measurements were taken using the BioTek Cytation Hybrid Multi-Mode Reader (Agilent Technologies).

### Human trial overview

**Figure 5:**
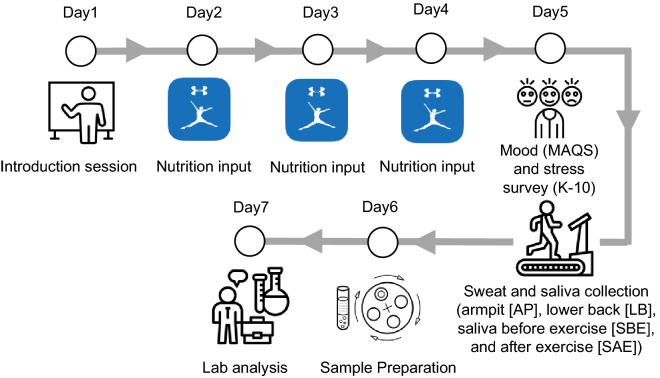
Research study diagram. A flowchart visualizing the steps that took place over the one-week span of the human trial experiments. The introductory meeting for all participants took place on the first day, followed by three days of nutritional data recording through the MyFitnessPal app, then the K10 survey was taken the morning of the exercise session on the fifth day. Day 6 involved the centrifugation process for sample preparation and the samples were analyzed with ELISA on day seven.

This research study was reviewed and approved by Binghamton University’s Institutional Review Board (IRB) with informed consent obtained from all participants prior to the start of the study. This study was advertised on campus. All methods were carried out in accordance with relevant guidelines and regulations. An initial meeting took place to discuss the study design with potential participants followed by a question-and-answer session. A total of 48 of healthy college students participated in this study. Dietary habits and water consumption were recorded for three days consecutive days through the MyFitnessPal mobile application for all participants for the study. Mood and stress were quantified using questionnaires taken by each participant prior to the exercise study session. The session was conducted between 10 a.m. and 12 p.m. to control confounding variables such as time of day and circadian rhythms of the participants. Participants initially provided 2 mL of saliva sample (SBE) using spitting techniques in 15 mL bio-reaction tubes. Participants then performed 30–60 min of aerobic cardio exercise by stationary cycling while wearing the sterilized medium gauze pads on their lower backs (LB) and armpits (AP). In this research study, obtained lower back sweat samples were labeled as *eccrine-dominant* and obtained armpit sweat samples were labeled as *apocrine-dominant* due to the presence of majority nonhair follicle-based and hair follicle-based sweat glands in these respective locations. Finally, participants provided 2 mL of saliva post-exercise (SAE) using spitting techniques in separate 15 mL bio-reaction tubes. Saliva samples were collected before and after exercise to quantify the effect of exercise intensity on cortisol secretion for participants in the study. The gauze pads were transferred to 50 mL centrifuge tubes, containing centrifuge grids, where the sweat was centrifuged out at 1060×*g* (3000 rpm) for 10 min to isolate sweat from the gauze pads^25^. Saliva samples were prepared by centrifugation at 1060×*g* for 15 min, stored for one hour at − 20 °C and then centrifuged once again at 1060×*g* at 15 minutes^26^. Figure 5 visualizes the entire human trial research study.

### Biochemical analysis

All spectroscopic analytical procedures followed the recommended protocols for the urinary cortisol ELISA and saliva cortisol ELISA kits’ manuals. A urinary cortisol ELISA kit was chosen for sweat cortisol determination as its unbound free cortisol range of detection, 0.47–200 ng/mL, is comparable to previously reported ranges of sweat cortisol^13,15^. Quantifiable biofluid samples were categorized into comparable and incomparable groupings. Comparable samples referred to whether an individual participant was able to provide yield quantifiable cortisol concentrations for the saliva and sweat samples they provided. Additionally, the responses of the ELISA reaction were tested with cortisol solutions in artificial sweat and water, confirming that urinary assay kits provided reliable results with sweat. A 1.0 mg/mL cortisol solution in methanol was diluted with methanol to a concentration of 1.0 μg/mL cortisol solution. This solution was then diluted further in both water and artificial sweat to make 0, 1, 5 and 30 ng/mL cortisol solutions for both solvents to compare against the cortisol calibrator solutions provided in urinary cortisol ELISA kit (Supplementary Fig. S1, Supplementary Table S2). Two separate calibration curves fitted with four parameter logistic curves (4PLC) allowed for quantitative analysis of cortisol in sweat and saliva (Supplementary Fig. S2).

### Nutritional value, stress and mood analysis

MyFitnessPal quantified macronutrient consumption (carbohydrates [g], protein [g], fat [g]), sugar consumption (g), and total caloric intake for participants in the study. Carbohydrates were subcategorized into simple and complex. This took into consideration each carbohydrate variation’s effect on blood glucose, as its correlation between stress cortisol secretion and blood glucose has been previously reported^27^. The average mass of nutrient consumption per day is used for data analysis. The AHEI measures diet quality in comparison to Dietary Guidelines for Americans, while also incorporating additional factors that focus on food consumption for chronic disease risk prediction^28^. AHEI scores were calculated using serving size recommendations of the five food groups (whole grains, protein, vegetables, fruit, and dairy) for each individual participant. A score of 10 was assigned for each category when the serving size for that food group was fulfilled, whereas if the requirements were not met, no points would be awarded for that category. Scores ranged from 0 to 50, where a score of 50 represented the healthiest eating pattern an individual could exhibit. BMR, the caloric energy (cal) required for homeostatic functioning at rest^29^, was calculated using the Mifflin-St. Jeor equation for each participant^30^. The caloric intake per day (Cal/Day), taken over the three-day span of nutritional tracking, was divided by the calculated BMR to calculate Cal per Day/BMR ratios.

The Kessler Psychological Distress Scale (K10), an extensively used 10-question examination to detect possible anxiety, depressive or mental distress-based disorders^31^, assessed individual mental stress levels. Each question requires individuals to assess how often they experience mental distress on a 5-Point Likert scale that ranges from 1 (i.e., none of the time) to 5 (i.e., all of the time)^32,33^. Participants took the K10 test the morning of the exercise study session, prior to sample collection. The K10 test categorizes the degree of distress numerically, where scores under 20 are likely to be well, scores from 20 to 24 are likely to have mild mental distress, scores from 25 to 29 are likely to have moderate mental distress, and scores from 30 to 50 are likely to have severe mental distress^34^. The low, mild, moderate and severe mental distress groups were all separated into cortisol concentration ranges, and possible significant differences were determined between groups. Participants also took a modified Mood and Anxiety Symptoms Questionnaire (MASQ) to assess their general distress and anxious arousal symptoms^35,36^. The MASQ is derived from tripartite model of anxiety and depression assessment, which separates mood into positive affect (PA) and negative affect (NA)^37^. PA is distinguished by positive emotional states that relate to overall mental wellness, whereas NA is characterized by aversive emotional states associated with symptoms of anxiety and depression^38^. The MASQ survey scores questions to a 5-Point Likert scale that range from 1 (i.e., no anxious symptoms felt at all) to 5 (i.e., anxious symptoms felt to the extreme). Participants responded to this questionnaire during the first morning of nutritional data collection and once again on the day of exercise study session. Similar to the K10, higher individual MASQ scores correlate to greater NAs of anxious symptoms on participants’ overall mood^39^.

### Statistical analysis

All collected biofluid cortisol and nutritional information populations were normalized using natural logarithm transformations (Supplementary Fig. S3)^40^ using Microsoft Excel. The raw biofluid cortisol concentration data was rightly skewed, which correlated with a previously conducted raw cortisol detection experimental results^41^. Statistical significance tests (independent t test, paired t test, ANOVA, and linear regression ANOVA) were able to be performed by using Microsoft Excel Data Analysis Toolpak and R software to compare biofluid cortisol samples and recorded dietary intake. An α significance level of 0.1 was used for all statistical analysis, as this pilot study compared various biofluid cortisol concentrations, numerous factors for dietary intake, multiple mood surveys, and a distress survey for a limited number of participants (n = 48). These varying factors contribute to underpowering this study and can deter from determining significant preliminary evidence when using a 0.05 α significance level^42–45^^.^

### Machine learning

Four machine learning algorithms, Decision Tree (DT), Random Forest, K-Nearest Neighbors (KNN), and Ordinal Regression were used to handle nonlinearity and complex structure of the dataset. Decision Tree algorithms rely on a tree-like model drawn upside down with its roots at the top, which does not require an extra process regardless of data type and multi-output. The Random Forest is also a robust algorithm that integrates DTs with good learning capability. The KNN is capable of predicting numerical values based on a similarity measure such as Euclidian distance or Hamming distance. Ordinal Regression denotes a family of statistical learning in which the goal is to predict a variable that is discrete and ordered (e.g., prediction of the rating).

For the training the models, three datasets were used to verify the impact on the cortisol assessment to predict the K10 score. First, the input dataset of 48 samples with 13 variables, which is a mixture of continuous [i.e., saliva before (ng/mL), BMI, Cal per day/BMR, Simple carbs (g), sugar (g), fat (g)] and discrete (i.e., sex, AHEI, age, recreational athlete, birth control) was constructed. Then, 28 comparable samples SBE (ng/mL) and AP (ng/mL) samples were selected to evaluate the predicative performance between the cortisol sampling types. Based on these 28 samples, two other input datasets are constructed: one containing the cortisol information from the saliva [i.e., SBE (ng/mL)] and another one is constructed by replacement of the cortisol from the saliva to that of armpit [i.e., AP (ng/mL)]. As a performance validation, leave-one-out cross validation is used, which is a special case of cross validation where the number of folds is same as the number of the sample. Therefore, the learning algorithm is applied once for each instance as a training set and using the selected instance as a single-item test set.

## Supplementary information

Supplementary Information

## References

[1] Baker LB, Wolfe AS (2020). Physiological mechanisms determining eccrine sweat composition. Eur. J. Appl. Physiol..

[2] Staufenbiel SM, Penninx BWJH, Spijker AT, Elzinga BM, van Rossum EFC (2013). Hair cortisol, stress exposure, and mental health in humans: A systematic review. Psychoneuroendocrinology.

[3] Dedovic K, Duchesne A, Andrews J, Engert V, Pruessner JC (2009). The brain and the stress axis: The neural correlates of cortisol regulation in response to stress. NeuroImage.

[4] Lupien SJ, Maheu F, Tu M, Fiocco A, Schramek TE (2007). The effects of stress and stress hormones on human cognition: Implications for the field of brain and cognition. Brain Cogn..

[5] Holsboer F, Ising M (2010). Stress hormone regulation: Biological role and translation into therapy. Annu. Rev. Psychol..

[6] Kaushik A, Vasudev A, Arya SK, Pasha SK, Bhansali S (2014). Recent advances in cortisol sensing technologies for point-of-care application. Biosens. Bioelectron..

[7] Torres SJ, Nowson CA (2007). Relationship between stress, eating behavior, and obesity. Nutrition.

[8] Lemmens SG, Born JM, Martens EA, Martens MJ, Westerterp-Plantenga MS (2011). Influence of consumption of a high-protein vs high-carbohydrate meal on the physiological cortisol and psychological mood response in men and women. PLoS One.

[9] Tsigos C, Chrousos GP (2002). Hypothalamic–pituitary–adrenal axis, neuroendocrine factors and stress. J. Psychosom. Res..

[10] Soriano-Rodríguez P (2010). Physiological concentrations of serum cortisol are related to vascular risk markers in prepubertal children. Pediatr. Res..

[11] Hellhammer DH, Wüst S, Kudielka BM (2009). Salivary cortisol as a biomarker in stress research. Psychoneuroendocrinology.

[12] Laudat MH (1988). Salivary cortisol measurement: A practical approach to assess pituitary-adrenal function. J. Clin. Endocrinol. Metab..

[13] Jia M, Chew WM, Feinstein Y, Skeath P, Sternberg EM (2016). Quantification of cortisol in human eccrine sweat by liquid chromatography–tandem mass spectrometry. Analyst.

[14] Grass J (2015). Sweat-inducing physiological challenges do not result in acute changes in hair cortisol concentrations. Psychoneuroendocrinology.

[15] Rice P (2019). CortiWatch: Watch-based cortisol tracker. Future Sci. OA.

[16] Wüst S (2000). The cortisol awakening response—normal values and confounds. Noise Health.

[17] Sogari G, Velez-Argumedo C, Gómez M, Mora C (2018). College students and eating habits: A study using an ecological model for healthy behavior. Nutrients.

[18] Hill EE (2008). Exercise and circulating Cortisol levels: The intensity threshold effect. J. Endocrinol. Invest..

[19] Fries E, Dettenborn L, Kirschbaum C (2009). The cortisol awakening response (CAR): Facts and future directions. Int. J. Psychophysiol..

[20] Jacks DE, Sowash J, Anning J, McGloughlin T, Andres F (2002). Effect of exercise at three exercise intensities on salivary cortisol. J. Strength Cond. Res..

[21] Cevada T, Vasques P, Moraes H, Deslandes A (2014). Salivary cortisol levels in athletes and nonathletes: A systematic review. Horm. Metab. Res..

[22] Tu E (2020). Comparison of colorimetric analyses to determine cortisol in human sweat. ACS Omega.

[23] MacMaster FP (2007). Development and sexual dimorphism of the pituitary gland. Life Sci..

[24] Torrente-Rodríguez RM (2020). Investigation of cortisol dynamics in human sweat using a graphene-based wireless mHealth system. Matter.

[25] Baker LB (2017). Sweat testing methodology in the field: Challenges and best practices. Gatorade Sports Sci. Inst..

[26] DCM020 Cortisol Saliva ELISA Assay Kit. *Cortisol Saliva ELISA Direct Immunoenzymatic Determination of Cortisol in Saliva.* https://www.eaglebio.com/content/DCM020_Cortisol%20Saliva_ELISA_Assay_Kit.pdf.

[27] Goiato MC (2019). Evaluation of the level of cortisol, capillary blood glucose, and blood pressure in response to anxiety of patients rehabilitated with complete dentures. BMC Oral Health.

[28] Al-Ibrahim AA, Jackson RT (2019). Healthy eating index versus alternate healthy index in relation to diabetes status and health markers in US adults: NHANES 2007–2010. Nutr. J..

[29] Sabounchi NS, Rahmandad H, Ammerman A (2013). Best-fitting prediction equations for basal metabolic rate: Informing obesity interventions in diverse populations. Int. J. Obes..

[30] Frankenfield D, Roth-Yousey L, Compher C (2005). Comparison of predictive equations for resting metabolic rate in healthy nonobese and obese adults: A systematic review. J. Am. Diet. Assoc..

[31] Baillie AJ (2005). Predictive gender and education bias in Kessler’s psychological distress Scale (k10). Soc. Psychiatry Psychiatr. Epidemiol..

[32] Easton SD, Safadi NS, Wang Y, Hasson RG (2017). The Kessler psychological distress scale: Translation and validation of an Arabic version. Health Qual. Life Outcomes.

[33] 5-Point Likert Scale. in *Handbook of Disease Burdens and Quality of Life Measures* (eds. Preedy, V. R. & Watson, R. R.) 4288–4288 (Springer New York, 2010). 10.1007/978-0-387-78665-0_6363.

[34] Kessler RC (2002). Short screening scales to monitor population prevalences and trends in non-specific psychological distress. Psychol. Med..

[35] Watson D (1995). Testing a tripartite model: I. Evaluating the convergent and discriminant validity of anxiety and depression symptom scales. J. Abnorm. Psychol..

[36] Lin A (2014). Validation of a short adaptation of the Mood and Anxiety Symptoms Questionnaire (MASQ) in adolescents and young adults. Psychiatry Res..

[37] Clark LA, Watson D (1991). Tripartite model of anxiety and depression: Psychometric evidence and taxonomic implications. J. Abnorm. Psychol..

[38] Wardenaar KJ (2010). Development and validation of a 30-item short adaptation of the Mood and Anxiety Symptoms Questionnaire (MASQ). Psychiatry Res..

[39] Light SN (2011). Reduced right ventrolateral prefrontal cortex activity while inhibiting positive affect is associated with improvement in hedonic capacity after 8 weeks of antidepressant treatment in major depressive disorder. Biol. Psychiatry.

[40] Duong M, Cohen JI, Convit A (2012). High cortisol levels are associated with low quality food choice in type 2 diabetes. Endocrine.

[41] Kobayashi H, Miyazaki Y (2015). Distribution characteristics of salivary cortisol measurements in a healthy young male population. J. Physiol. Anthropol..

[42] Wood J, Lambert M (1999). Sample size calculations for trials in health services research. J. Health Serv. Res. Policy.

[43] Julious SA, Patterson SD (2004). Sample sizes for estimation in clinical research. Pharm. Stat..

[44] Lee EC, Whitehead AL, Jacques RM, Julious SA (2014). The statistical interpretation of pilot trials: Should significance thresholds be reconsidered?. BMC Med. Res. Methodol..

[45] Kianifard F, Islam MZ (2011). A guide to the design and analysis of small clinical studies. Pharm. Stat..

