# Supplementary material for: Sweat and saliva cortisol response to stress and nutrition factors
Source: Sci Rep. 2020 Nov 4;10:19050. doi: 10.1038/s41598-020-75871-3 (PMC7643128; doi:10.1038/s41598-020-75871-3)
Supplement: Supplementary file 1 — Supplementary Information [file 41598_2020_75871_MOESM1_ESM.docx]

**Supplementary Dataset for**

**Sweat and Saliva Cortisol Response to Stress and Nutrition Factors**

Paul Pearlmutter^a^, Gia Derose^b^, Cheyenne Samson^a^, Nicholas Linehan^a^, Yuqiao Cen^c^, Lina Begdache^b^, Daehan Won^c^ Ahyeon Koh^a^*

^a^ Department of Biomedical Engineering, Binghamton University-State University of New York, Binghamton, NY 13902

^b^ Health and Wellness Studies Department, Binghamton University-State University of New York, Binghamton, NY 13902

^c^ Department of System Sciences and Industrial Engineering, Binghamton University-State University of New York, Binghamton, NY 13902

* Corresponding Author: [akoh@binghamton.edu](about:blank); Tel +1 (607) 777-5422; Fax +1 (607) 777-5780

**Figures and Tables**

**Supplementary Table S1.** Comparison of age ranges for different participant subgroups.

**Supplementary Figure S1.** Comparison of urinary cortisol ELISA concentration curves.

**Supplementary Table S2.** Urinary ELISA absorbance values for cortisol concentrations in various solutions.

**Supplementary Figure S2.** Cortisol concentration ELISA calibration curves.

**Supplementary Figure S3.** Natural log transformations of biofluid datasets.

**Supplementary Figure S4.** Natural logarithm biofluid cortisol concentration ranges for participant subgroups.

**Supplementary Figure S5.** Total biofluid cortisol concentration ranges based on K10 score groupings.

**Supplementary Figure S6.** Comparison chart for actual and predicted K10 score of individual participants.

**Figures and Tables**

**Supplementary Table S1.** Comparison of age ranges for different participant subgroups.

|  | **Male Athletes** | **Male**  **Non-Athletes** | **Female Athletes** | **Female**  **Non-Athletes** |
| --- | --- | --- | --- | --- |
| **Average** | 21.5 | 23.9 | 21.0 | 22.7 |
| **Standard Deviation** | 3.7 | 4.0 | 2.6 | 2.2 |
| **Range** | 19-29 | 20-31 | 19-30 | 21-29 |
| **Total Participants** | 6 | 12 | 16 | 14 |

**Supplementary Figure S1. Comparison of urinary cortisol ELISA concentration curves.** Cortisol concentration values in **(A)** urinary calibrator solution, **(B)** water, and **(C)** artificial sweat.

**Supplementary Table S2.** Urinary ELISA absorbance values for cortisol concentrations in various solutions

|  | **0 ng/mL** | **1 ng/mL** | **5 ng/mL** | **30 ng/mL** |
| --- | --- | --- | --- | --- |
| **Calibrator** | 3.21 ± 0.02 | 2.41 ± 0.12 | 1.356 ± 0.01 | 0.528 ± 0.01 |
| **Water** | 2.92 ± 0.09 | 2.63 ± 0.05 | 1.89 ± 0.10 | 0.72 ± 0.05 |
| **Artificial Sweat** | 2.75 ± 0.09 | 2.41 ± 0.09 | 1.65 ± 0.05 | 0.76 ± 0.02 |
| **Coefficient of Variation (%)** | 7.9 | 5.1 | 16.3 | 18.6 |

The values signify mean ± standard deviation. Coefficient of variation is calculated between all 3 solutions at each concentration value.

**Supplementary Figure S2. Cortisol concentration ELISA calibration curves.** 4PLC curves for **(A)** sweat cortisol (R^2^ = 1) **(B)** saliva cortisol (R^2^ = 0.9996).

**Supplementary Figure S3. Natural log transformations of biofluid datasets. (A)** SBE (Raw: Shapiro-Wilks: *p* < 0.05, normal: Shapiro-Wilks: *p* > 0.05) **(B)** AP (raw: Shapiro-Wilks: *p* < 0.05, normal: Shapiro-Wilks: *p* > 0.05) **(C)** SAE (raw: Shapiro-Wilks: *p* < 0.05, normal: Shapiro-Wilks: *p* > 0.05) **(D)** LB (raw: Shapiro-Wilks: *p* > 0.05, normal: Shapiro-Wilks: *p* > 0.05).

**Supplementary Figure S4. Natural logarithm biofluid cortisol concentration ranges for participant subgroups. (A)** No significant differences determined between athlete (M: n = 4, F: n = 11) or non-athlete (M: n = 4, F: n = 6) sexes for AP cortisol levels. Comparable SBE-SAE levels between athletes (M: n = 4, F: n = 15) and non-athletes (M: n = 11, F: n = 9) for **(B)** males and **(C)** females. Significant decrease in saliva cortisol levels after exercise was prevalent for non-athlete males (two-tailed paired t-test, *p* = 0.039) and non-athlete females (two-tailed paired t-test, *p* = 0.080).

**Supplementary Figure S5. Total biofluid cortisol concentration ranges based on K10 score groupings.** No significant differences in cortisol levels found between low (10-19), mild mental distress (20-24), moderate mental distress (25-29), and severe mental distress (30+) groups for **(A)** SBE **(B)** SAE **(C)** AP


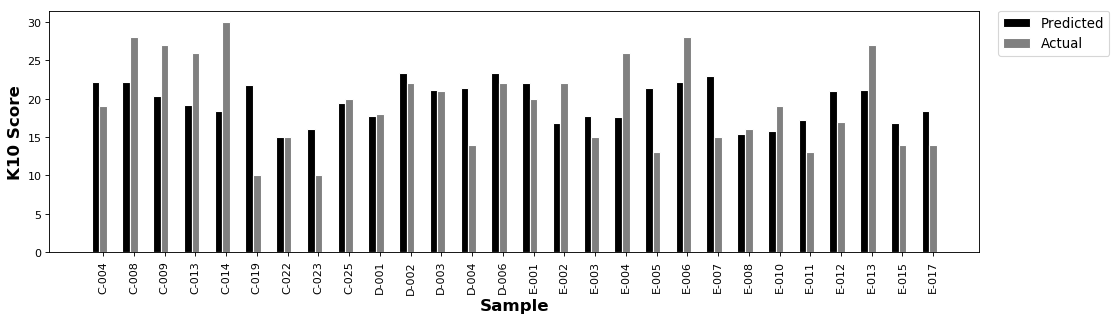


**Supplementary Figure S6.** Comparison chart for actual and predicted K10 score of individual participants.
